# Development of a conceptual model of intertemporal decision-making ability for young and middle-aged stroke patients within physical activity: a qualitative study

**DOI:** 10.3389/fpsyg.2026.1877769

**Published:** 2026-07-20

**Authors:** Xingru Li, Chao Zhang, Yidan Liu, Ping Zhou

**Affiliations:** 1 School of Nursing, Jiangxi Medical College, Nanchang University, Nanchang, Jiangxi, China; 2 Department of Nursing, The Second Affiliated Hospital, Jiangxi Medical College, Nanchang University, Nanchang, Jiangxi, China

**Keywords:** conceptual model, intertemporal decision-making, physical activity, qualitative research, stroke

## Abstract

**Background:**

Intertemporal decision-making ability is critical for behavior change and maintenance. While a conceptual model of this ability exists for chronic patients, how it manifests in the physical activity (PA) context among young and middle-aged stroke patients is unclear. Therefore, this study aimed to contextually validate and extend the generic conceptual model, and to explore its applicability to PA among young and middle-aged stroke patients.

**Methods:**

This qualitative study employed semi-structured interviews with 16 stroke patients, who were purposively sampled from a hospital in Nanchang. Data were analyzed using directed content analysis.

**Results:**

The conceptual model comprises four dimensions: (1) impulse control (including impulse awareness and inhibition of immediate impulses); (2) emotional self-regulation (including emotional awareness and application of emotion regulation strategies); (3) future health valuation (including perceived long-term benefits of PA and the value attributed to family roles and social responsibilities); (4) future-oriented thinking and planning (including future scenario imagination, construction of causal chains, and shortening of temporal distance).

**Conclusion:**

The intertemporal decision-making ability of young and middle-aged stroke patients during PA exhibits multidimensional characteristics, including impulse control, emotional self-regulation, future health valuation, and future-oriented thinking and planning. Family and social responsibilities, along with warning imagery, endow these dimensions with unique connotations that extend beyond the general model. Future research should develop specific measurement tools based on the dimensional structure of this ability and design personalized intervention programs.

## 1 Introduction

Stroke is the second leading cause of death and the third leading cause of disability worldwide. This disease not only poses a threat to individual health and family well-being but also exerts a profound impact on the sustainable development of the socioeconomy (GBD 2019 Stroke Collaborators, 2021). Notably, the age of onset of stroke is continuously shifting earlier, with a marked trend toward younger populations. It has been reported that stroke affects more than 15 million people annually, including at least 1.5 million young individuals (Ekker et al., 2019).

A previous study has shown that 94.3% of stroke events are closely associated with modifiable risk factors (Wang et al., 2022), among which physical activity (PA), defined as any bodily movement produced by skeletal muscle contraction that results in energy expenditure, represents the most critical modifiable risk factor (Guo et al., 2022) and accounts for a large proportion of strokes in young and middle-aged adults (Abdalla et al., 2018). The World Health Organization’s Guidelines (Bull et al., 2020) recommend that adults with chronic conditions should engage in 150–300 min of moderate-intensity aerobic PA per week. Furthermore, the guidelines also emphasize that PA is essential for reducing disability and improving physical function, walking ability, and balance after stroke (Roaldsen et al., 2022). Despite this, many stroke patients do not meet the recommended PA levels compared to their healthy peers (English et al., 2016).

Numerous factors contributing to low levels of PA among stroke patients have been identified, including personal, environmental, and motivational factors (Moore et al., 2022; Nicholson et al., 2013; Peltonen et al., 2025). However, little attention has been paid to the influence of temporal factors on PA participation and maintenance, specifically the trade-off that patients make between immediate effort and long-term benefits. When deciding whether to engage in PA, individuals often prefer behaviors that provide immediate gratification, such as remaining sedentary for comfort, over health behaviors that yield greater future benefits, such as engaging in PA to prevent disease. This choice process constitutes intertemporal decision-making (Jin et al., 2024). When an individual can weigh immediate and future benefits while demonstrating a future-oriented decision-making preference, they are considered to possess intertemporal decision-making ability (Wang et al., 2024). This ability plays a key role in behavior maintenance and change, and serves as a useful predictor of adherence to health behaviors (including PA) and clinical outcomes (Bickel et al., 2012; Lebeau et al., 2016; Shao et al., 2026) as well as a promising intervention target (Sheffer et al., 2018; Vaughn et al., 2021) in patients with chronic conditions.

In stroke patients, brain damage, particularly to the frontal lobe or striatum, can lead to declines in executive function and emotional disorders, which may further impair intertemporal decision-making ability and exacerbate the difficulty of behavior change (Dixon et al., 2005). Furthermore, compared with older adults, young and middle-aged individuals may exhibit less stable impulse inhibition and delayed gratification, leading them to prefer immediate rewards over long-term health benefits (Frederick et al., 2002). Although young and middle-aged individuals have a longer future time perspective, their youth or limited experience may lead to weaker self-regulation and future orientation during transitions to major life events (e.g., illness), further undermining their intertemporal decision-making ability (de Ridder et al., 2012).

It is necessary to differentiate between the concepts related to intertemporal decision-making ability. For example, intertemporal decision-making refers to an individual’s choice among options that occur at different points in time (Frederick et al., 2002), emphasizing the underlying behavioral tendency or phenomenon. Delay discounting is a key feature of intertemporal decision-making, referring to the degree to which future gains or losses are discounted as a function of time delay (Castellanos-Ryan et al., 2016). A related concept is future orientation, which refers to an individual’s ability to anticipate and plan for future consequences prior to taking action, with an emphasis on establishing long-term goals (Apouey, 2018). These concepts are related to, but not synonymous with, intertemporal decision-making ability, as the latter comprises multiple psychological dimensions, including cognitive control, emotion regulation, impulse inhibition, and the weighting of uncertainties (Kókai et al., 2026). The quantitative indicator derived from intertemporal choices-the delay discounting rate (*k*-value)-is considered a valid indicator of intertemporal decision-making ability (He and Li, 2020). However, this parameter captures a phenomenon rather than the ability itself. Furthermore, the delay discounting rate is based on monetary choices and cannot capture the multidimensional structure of intertemporal decision-making ability in specific health contexts.

A previous study (Li et al., 2026) explored a conceptual model of intertemporal decision-making ability in patients with chronic conditions. The model comprised four dimensions: impulse control, emotional self-regulation, future health valuation, and future-oriented thinking and planning. However, this general model was developed based on multiple chronic diseases and various health behavior contexts, its applicability to PA among young and middle-aged stroke patients remains unclear. Therefore, this study conducted semi-structured in-depth interviews with young and middle-aged stroke patients, followed by a directed content analysis. The findings revealed how dimensions from a generic model manifest specifically in the context of PA. Additionally, new elements, such as the value attributed to family and social responsibilities, and warning imagery, were identified, thereby enriching the original model. The conceptual model of intertemporal decision-making ability in the context of PA was expected to inform the development of targeted assessment tools and interventions.

## 2 Methods

### 2.1 Study design

Based on the previously constructed conceptual model of intertemporal decision-making ability in chronic disease patients (Li et al., 2026), semi-structured in-depth interviews with directed content analysis were employed to explore the multidimensional structure and manifestations of intertemporal decision-making ability in young and middle-aged stroke patients within the PA context. Directed content analysis, also referred to as deductive category development, is commonly employed to validate or extend an existing conceptual framework or theory. This approach is particularly suitable for studies in which prior theory or literature on the phenomenon of interest is already available (Hsieh and Shannon, 2005). The research report adheres to the Consolidated Criteria for Reporting Qualitative Research (COREQ) (Tong et al., 2007).

### 2.2 Setting and participants

Purposive sampling was used to recruit young and middle-aged stroke patients living at home with diverse demographic characteristics, disease types, and durations from the stroke health management nursing clinic at a tertiary hospital in China to enhance the sample’s representativeness. The inclusion criteria were as follows: (1) confirmed clinical diagnosis of stroke; (2) disease duration over 6 months, with current residence at home. This time point was selected because patients were considered to have passed the acute phase after 6 months (Bernhardt et al., 2017), thereby avoiding the effects of marked emotional fluctuations on intertemporal decision-making ability; (3) aged 18–59 years; (4) modified Rankin Scale (mRS) ≤2, ensuring basic independent mobility and discussions ability related to PA; (5) no cognitive or language impairments. Patients with severe mental illnesses, as well as those with concomitant severe cardiac, pulmonary, hepatic, or renal insufficiency, or malignant tumors, were excluded.

### 2.3 Data collection

The researchers first contacted the manager of the stroke health management nursing clinic to explain the study’s purpose and procedures. During routine clinic visits, the manager approached potential participants, provided them with information about the study’s objectives and methods, and obtained their preliminary consent. Subsequently, the manager referred the contact information of these individuals to the principal investigator (LXR). Before each interview, LXR contacted the potential participants, described the study protocol with particular emphasis on privacy protection, obtained their electronic informed consent, and scheduled the interview time. The interview guide was consistent with the previously published conceptual model of intertemporal decision-making ability in patients with chronic diseases (Li et al., 2026). Before finalizing the interview guide, pre-interviews were conducted with three participants, leading to revisions and the addition of prompts such as “Could you elaborate on that?” and “Could you provide an example?” The data from the pre-interviews were not analyzed. The main interview guide is presented in Table 1. One-on-one video interviews were conducted in a quiet, private room. The main interviews were led by LXR, while another researcher (LYD) was present during the interviews, primarily documenting participants’ tone and non-verbal cues and monitoring the lead interviewer for potentially suggestive language. All interviews were audio-recorded, with each session lasting 30–45 min. The sample size was determined by data saturation, defined as the point at which no new themes emerged from consecutive interviews (Rahimi and Khatooni, 2024). After the 14th interview, preliminary analysis by two researchers confirmed that no new themes or subthemes emerged. To further verify saturation, two additional interviews were conducted, and analysis showed that no new information was obtained.

**Table 1:** The main interview outline.

Dimension	Definition of dimension (Li et al., 2026)	Questions
Impulse control	The ability of patients to control impulsive responses when faced with competing temptations for PA.	Under what circumstances do you experience the urge to “stop moving” during PA? Can you provide an example?
		What do you think causes these urges?
		What actions do you take to suppress these urges?
Emotional self-regulation	The ability of patients to identify and regulate the interference of negative emotions on PA decision-making.	When faced with PA, what negative emotions do you experience?
		When faced with negative emotions, how do you regulate them?
Future health valuation	The ability of patients to assign high subjective value to future health outcomes of PA.	What benefits do you think participating in or adhering to PA will have on your future health?
Future-oriented thinking and planning	The ability of patients to direct their thinking toward the future, set goals, and make plans.	Do you imagine future scenarios after persisting in PA? Can you describe them in detail?
		What PA plans or goals do you set for yourself? What role do they play?

### 2.4 Data analysis

Participants’ basic information was anonymized. Audio recordings were transcribed verbatim by a professional transcription service within 24 h, and LXR verified the transcripts. A directed content analysis approach was used to analyze the data (Hsieh and Shannon, 2005). First, LXR and LYD repeatedly read the transcripts to familiarize themselves with the data. During this stage, the two authors documented their initial impressions. Second, the four dimensions of the conceptual model of intertemporal decision-making ability in patients with chronic diseases (Li et al., 2026), namely impulse control, emotional self-regulation, future health valuation, and future-oriented thinking and planning, were predefined as initial coding categories. Two researchers independently identified and coded meaningful text segments relevant to these categories, and temporarily assigned these codes to the most relevant categories. However, to avoid confirmation bias, the researchers remained open to data that did not fall into the predefined categories. Inductive coding was applied to text that could not be assigned to the predefined categories, in order to identify potential new themes. Following independent coding of five interviews, the two researchers held a discussion meeting to resolve discrepancies between the codes and their corresponding categories. In total, three discussion meetings were conducted. Any disagreements were resolved through discussion with the corresponding author, ZC. Subsequently, all codes under each category were clustered and analyzed to identify subthemes reflecting the specific content within each dimension. Following the three discussion sessions, all team members convened to discuss the coherence and logical consistency of the codes, subthemes, and themes. The combination of deductive and inductive coding ensured that the findings were both theoretically grounded and rooted in the language of young and middle-aged stroke patients, thereby enhancing the practical applicability of the findings (Read et al., 2026). All meaningful texts were incorporated into the four dimensions after analysis. Although sub-themes not explicitly described in the original model emerged under some dimensions (e.g., “warning imagery”), these were considered contextual elaborations of the existing dimensions. To enhance the transparency of the analysis, coding examples were provided in Supplementary file 1. The final results were presented to the participants, who provided no further suggestions.

### 2.5 Rigor and reflexivity

This study adhered to Lincoln and Guba’s standards (Lincoln and Guba, 1988). The researchers’ repeated immersion in transcripts and member checking enhanced credibility; peer debriefing and meeting discussions increased dependability; the combination of interview transcripts and field notes strengthened confirmability; and providing detailed descriptions of participant characteristics in the methodology and maximizing the use of participants’ original language in the findings promoted transferability. Furthermore, reliance on an interview guide structured around predefined categories may constrain the emergence of novel content. This bias can be mitigated by employing open-ended questions and consciously refraining from forcing interview text into these predefined categories.

The first author is doctoral student in nursing with extensive experience in qualitative research and has 2 years of experience working in a rehabilitation department. The second author has over 10 years of experience in neurosurgery and is currently a doctoral supervisor and nursing manager. None of the authors had prior acquaintance with the participants. The researchers’ clinical work experience informed the data analysis but may have introduced potential over-interpretation bias. To mitigate these potential biases, all researchers were asked to reflect on their identities throughout the analysis. For example, regarding the code “mobilizing past memories to suppress impulses,” some team members reflected that, due to their familiarity with the field of memory cognition, they tended to assign it to the dimension of “future-oriented thinking and planning.” However, after re-examining the participants’ original quotations and engaging in team discussions, the code was considered to be associated with resisting the impulse to give up PA in the present moment, and was ultimately classified under the dimension of “impulse control”.

### 2.6 Ethical considerations

This study followed the principles of the Declaration of Helsinki. Participants were informed of their right to withdraw from the study at any time without any negative consequences. Electronic written informed consent was obtained from all participants. The identities of the participants were anonymized at all stages. Only the research team had access to the password-protected computer containing the data. The study protocol was approved by the Ethics Committee of the Second Affiliated Hospital of Nanchang University (approval number: O-Medical Research Ethics Review [2026] No. 68).

## 3 Results

**Figure 1:**
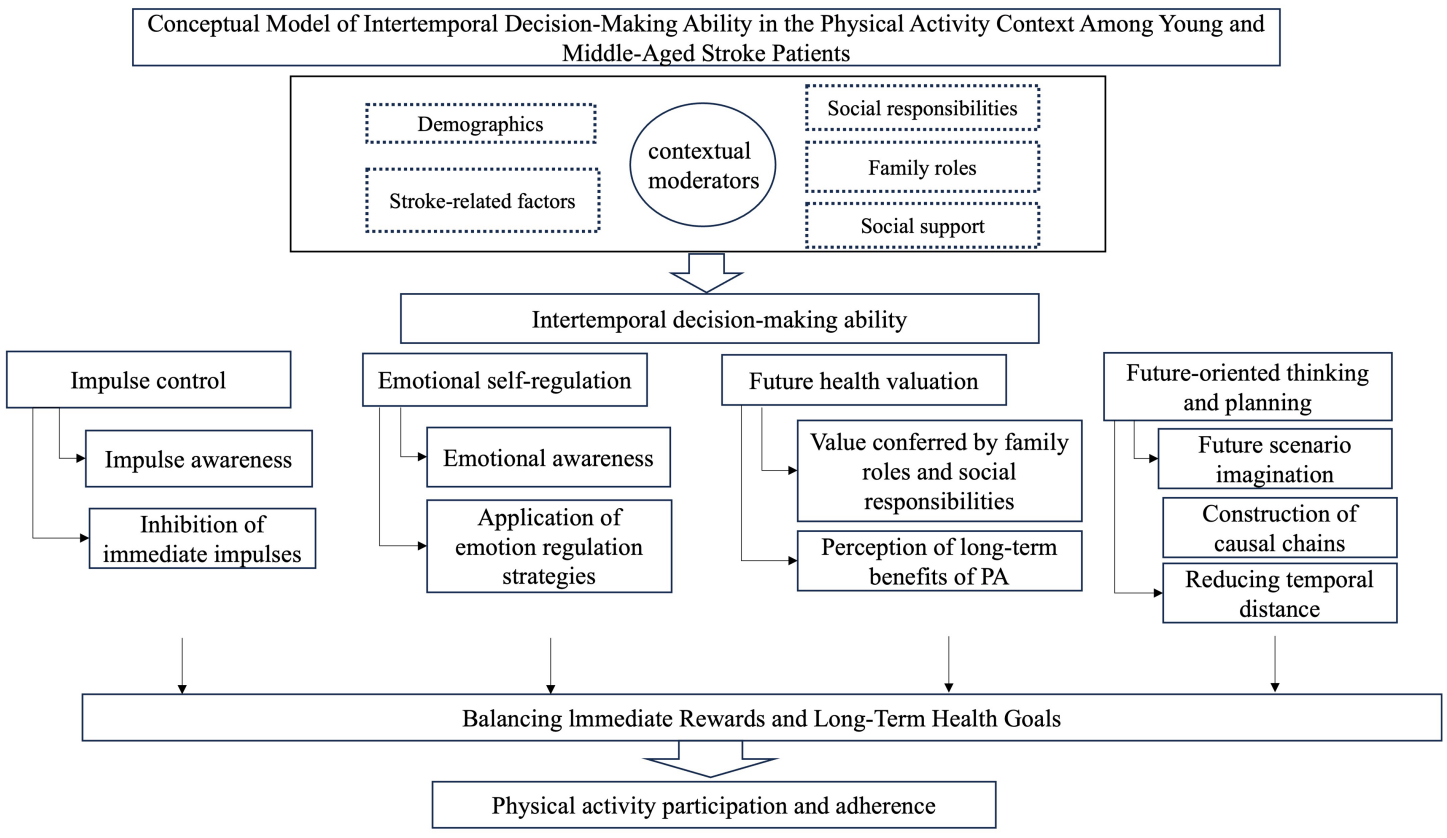
Conceptual model of intertemporal decision-making ability in the physical activity context among young and middle-aged stroke patients.

Sixteen participants were enrolled in this study, including 10 patients with ischemic stroke and 6 with intracerebral hemorrhage. The disease duration ranged from 7 months to 6 years. Detailed demographic characteristics are presented in Table 2. Following data analysis, a conceptual model of intertemporal decision-making ability in stroke within the PA context was identified, as shown in Figure 1.

**Table 2:** Demographic characteristics.

ID	Gender	Age (years)	Educational level	Marital status	Employment	Residence	Living arrangement	Disease type	Disease duration	mRS
P1	Female	32	Junior college	Unmarried	No	Urban	Living alone	Ischemic stroke	10 months	2
P2	Female	32	Master’s degree	Unmarried	Yes	Urban	Living alone	Ischemic stroke	2 years	1
P3	Female	32	Bachelor’s degree	Unmarried	Yes	Urban	Living alone	Ischemic stroke	2 years	1
P4	Female	41	Bachelor’s degree	Married	No	Urban	With family	Intracerebral hemorrhage	2 years	2
P5	Male	56	Junior college	Unmarried	Yes	Rural	With family	Ischemic stroke	3 years	1
P6	Male	47	Junior college	Married	Yes	Urban	With family	Intracerebral hemorrhage	6 years	1
P7	Female	34	Bachelor’s degree	Unmarried	Yes	Urban	Living alone	Intracerebral hemorrhage	5 years	2
P8	Male	57	High school	Married	No	Rural	With family	Ischemic stroke	3 years	0
P9	Male	47	Bachelor’s degree	Married	No	Urban	With family	Ischemic stroke	3 years	0
P10	Female	25	Bachelor’s degree	Unmarried	No	Urban	With family	Ischemic stroke	1 year	0
P11	Male	59	Primary school	Married	No	Rural	With family	Ischemic stroke	2 years	1
P12	Female	22	Junior college	Unmarried	No	Urban	With family	Ischemic stroke	4 years	2
P13	Male	30	Bachelor’s degree	Married	No	Urban	With family	Ischemic stroke	1 year	0
P14	Male	31	Primary school	Divorced	No	Urban	With family	Intracerebral hemorrhage	7 months	1
P15	Female	39	Bachelor’s degree	Married	Yes	Urban	With family	Intracerebral hemorrhage	9 months	1
P16	Female	31	Master’s degree	Married	Yes	Urban	With family	Intracerebral hemorrhage	1 year	2

### 3.1 Theme 1: impulse control

#### 3.1.1 Subtheme 1: impulse awareness

Impulse awareness refers to a participant’s ability to recognize an impulse when it arises and identify its source.

Participants can identify the main causes of the impulse to discontinue PA, including adverse weather, physical fatigue, a preference for comfort, and monotonous forms of exercise. Participants identified fatigue as a common and distinctive symptom that generated a strong urge to interrupt PA. *“I can usually only persist for 20 min, and then I feel tired and cannot continue”* (P1). Patients with milder symptoms who were employed expressed that their busy work schedules caused them to no longer think about PA participation. *“I have to say that I am usually very busy at work, and after coming home, I just want to lie down”* (P3). The availability of leisure and entertainment in the home environment (e.g., sofas, televisions, mobile phones) also frequently led to inactivity. P1 further added, *“Sometimes when I’m at home scrolling through videos or watching TV and see something I like, I just do not feel like moving anymore.”* One participant reported that monotonous PA programs diminished their interest in engaging in PA, ultimately leading to exercise cessation. *“The most difficult thing is the monotony and repetition. You lift your legs every day, you walk, it is that repetitive process that makes you not want to do it”* (P6).

#### 3.1.2 Subtheme 2: inhibition of immediate impulses

Inhibition of immediate impulses refers to participants actively adopting specific behavioral strategies to reduce the intensity of an impulse or to bypass it.

*Setting rewards*: to overcome the impulse to avoid PA, some participants established immediate self-reward mechanisms. They deconstructed the long-term health benefits of sustained PA into immediate incentives, thereby effectively counteracting inactivity. *“I tell myself, if I complete 80 reps today, I’ll give myself a reward, something I’ve wanted to eat but have not, or something I’ve wanted to play but have not”* (P12).

Maintaining activity baseline: some participants reported that when experiencing fatigue, they would adjust the performance threshold for PA promptly to maintain basic engagement, rather than completely giving up. As P8 stated, *“If I feel very tired today, I will adjust my amount of exercise, as long as it keeps me in an active state.”* Some participants further added that the impulse to “pause PA” peaks in the middle of the exercise session, and they often replace the original high-intensity activity with a lower-intensity activity (such as walking). *“When my body starts to feel tired, I will stop and switch to walking”* (P4).

Leveraging memory and experience: participants often drew on past positive exercise experiences or the success stories of others to counteract inertia. P9 reported, *“I thought of my classmate, who also had a cerebral infarction; he recovered very well, which is one of my motivations to persist in PA.”* Some participants created “memory videos” to document daily changes resulting from their PA participation. When hesitant about engaging in PA, they reviewed their trajectory of progress to reshape their beliefs about PA. *“I make some videos to review the progress I have made. When I do not want to exercise, I take them out and watch them; I know I will get better.”* (P4).

Self-reassurance: some participants indicated that they develop a self-reassurance mechanism when experiencing “exercise hesitation.” For example, P5 described externalizing the internal reassurance process as a dialogue with an “angel”: *“When I do not want to go out, my angel will come out and persuade me to exercise, just for a little while”*.

### 3.2 Theme 2: emotional self-regulation

#### 3.2.1 Subtheme 1: emotional awareness

Emotional awareness refers to participants’ ability to recognize and distinguish the negative emotions they experience when facing PA, as well as their sources.

Some participants can clearly identify the emotional sources underlying their unwillingness to engage in PA. They frequently reported that body image shame during PA, frustration over slow rehabilitation progress, and feelings of illness uncertainty triggered an impulse to pause PA. P15 reported, *“My limbs are not like before; I even walk with a limp.”* P5 indicated that a plateau in exercise leads to anxiety, which in turn triggers the impulse to *rest*: *“If I exercise for one or two weeks and see no results, I feel anxious and have thoughts of not wanting to exercise.”* P10 expressed that illness uncertainty makes her hesitant when making exercise decisions: *“I do not know whether my condition allows aerobic or anaerobic exercise, I’m afraid my movements might affect my disease progression”*.

#### 3.2.2 Subtheme 2: application of emotion regulation strategies

Attention shifting: many participants redirected their attention from negative emotions to the movements of PA by listening to music and thinking. *“I listen to music to distract myself. That is, I focus on the movements rather than on my emotions, and that makes me feel better”* (P1).

Seeking social support: seeking encouragement and empathetic listening from family and friends was mentioned as helpful in alleviating negative emotions. Notably, professional psychological counseling was also mentioned but received mixed evaluations. Some participants believed that *“psychological counselors are not very effective; what really matters is overcoming it on your own”* (P6), whereas others strongly recommended that healthcare professionals, especially rehabilitation therapists, should prioritize patients’ psychological rehabilitation, a need that they felt is often overlooked by these professionals. As P8 reported, *“Many rehabilitation therapists think that only physical rehabilitation counts as real recovery… this is a bias in rehabilitation”*.

*Cognitive restructuring*: some participants were able to change their interpretation of negative events (e.g., body image, falls). Body image disturbance caused participants to experience social shame during PA. Some participants reshaped their cognition through positive psychological self-suggestion. *“Since they are all looking at me anyway, I will present myself in the best possible appearance”* (P12). Patients with more severe illnesses often experienced falls during PA in the early stages of rehabilitation, whereas P12 attributed a positive meaning to falls, believing that each fall symbolizes “progress”.

*Self-acceptance and self-compassion*: some participants gradually accepted the changes in limb function during PA; they no longer engaged in self-condemnation but instead demonstrated self-compassion. P8 stated, *“It is just that my walking looks awkward; I have not done anything wrong.”* P16 also stated, *“I no longer pursue perfection; at least I can now live independently”*.

*Utilizing the immediate benefits of exercise*: some participants acknowledged the immediate efficacy of PA in countering negative emotions. This immediate positive feedback formed a positive self-reinforcing cycle, helping patients overcome initial inertia and negative emotions. As P2 stated, *“Exercise itself brings me a lot of joy and fun. If I am in a bad mood, I just go and exercise”* (P2).

### 3.3 Theme 3: future health valuation

#### 3.3.1 Subtheme 1: perception of long-term benefits of PA

Most participants reported that the perceived association between current PA participation and future benefits was primarily reflected in reduced stroke recurrence risk and improved recovery of limb function. P1 stated, *“I never thought about giving up exercise. If I… my physical function might deteriorate. To get better, I definitely need to exercise.”* The delayed nature of the future benefits of PA often led participants to question its effectiveness. Some participants reported that by consulting relevant literature and seeking advice from professionals, they came to understand that the benefits of PA participation arise from *“quantitative change leading to qualitative change”*.

#### 3.3.2 Subtheme 2: value conferred by family roles and social responsibilities

Young and middle-aged participants are at a life stage when they have to support both aging parents and young children. Some male participants reported that adhering to PA helped them fulfill their duty of caring for their elderly parents and avoid becoming a burden to their family. Most male participants were the primary breadwinners of their families and further linked the value of PA to returning to work. As P9 stated, *“I need to keep exercising. At our age, no boss wants an employee who walks with a limp.”* Additionally, some female participants linked the value of sustained PA to fulfilling their maternal role. *“Because my hands and feet are not very agile, my child has become sensible. Sometimes when I look at her, my heart aches. That’s why I cannot give up exercising, she is still so young.”* (P15).

### 3.4 Theme 4: future-oriented thinking and planning

#### 3.4.1 Subtheme 1: future scenario imagination

Some participants can develop specific, positive future scenarios for sustained PA participation. For example, P9 imagined becoming a sports blogger: *“I imagine myself becoming an internet celebrity. After all, I keep posting running-related videos every day, and many people send me private messages.”* P6 imagined participating in a marathon again after persisting with exercise. *“I imagine myself returning to the marathon track; this gives me the motivation to keep going.”* P8 also stated that imagining a positive future instilled a sense of hope, thereby effectively overcoming inertia and actively engaging in PA. *“Imagining the future gives me hope. I imagine myself returning to my previous state through exercise and going back to my former job.”* (P8) It is worth noting that patients with poorer limb function often engaged in “cautionary imagery.” For example, P7 imagined a future scenario in which he was lying in bed, unable to care for himself, and P5 imagined his elderly mother shedding tears. Although they perceived such imagery as a motivator for engaging in PA, it may have potential negative effects on mental health over the long term.

#### 3.4.2 Subtheme 2: construction of causal chains

Some participants linked a future healthy and active self-image to PA and described the causal relationship between them. P12 stated, *“In my mind, I have an image of my future self, a very healthy, energetic, and strong person. Being healthy and energetic means I should exercise.”* This causal chain transformed abstract future benefits into a perceptible action pathway. P6 stated, *“In my imagination, I unconsciously ask myself, ‘To achieve that imagined state, what do I need to do now’”*?

#### 3.4.3 Subtheme 3: reducing temporal distance

Some participants perceived the future as very distant and preferred setting small or short-term goals rather than long-term ones. This essentially represents an adaptive adjustment to future time discounting. When the psychological distance of future benefits is too far, individuals tend to set short-term goals to reduce the temporal distance. *“I can set short-term goals, such as what I need to do every day. Long-term goals are useless for me because they are too far away.”* (P2) P6 visualized the plan, thereby bringing the future closer: *“I have memos on my phone; I write down the plan, and every day I am getting closer to it”*.

## 4 Discussion

Compared with the general model for chronic disease patients proposed by Li et al. (2026), this study provides specific contextual information and unique characteristics of intertemporal decision-making ability in PA among young and middle-aged stroke patients. Novel findings, such as that family and social responsibility endow PA with future value and that cautionary imagination serves as a source of motivation, reflect the view of Attema et al. (2018) that intertemporal decision-making in the health domain is more complex than in the monetary domain. A single parameter (i.e., the delay discounting rate) is insufficient to capture its full connotation. The findings are expected to provide a foundation for the subsequent development of specific measurement tools and targeted interventions.

This study reveals that participants with better intertemporal decision-making ability can consciously recognize the impulse and its source of “unwillingness to engage in PA,” and spontaneously develop impulse inhibition strategies. This process reflects the top-down regulation of the cognitive control system over the impulse favoring immediate comfort within the dual-system theory framework (Evans, 2008).

Furthermore, these self-reported strategies from participants (e.g., self-reward, maintaining an activity baseline, and mobilizing experiential strategies) share common features with behavioral economic approaches, such as incentive strategies, commitment devices, and peer norms (Saposnik et al., 2026). However, their effectiveness in improving intertemporal decision-making ability or PA levels in this population remains to be validated in future empirical research.

Although participants employed various strategies to alleviate negative emotions, views on the role of social support in regulating them remain inconsistent. Mental health is considered a neglected aspect by professionals such as rehabilitation therapists, and patients’ psychological support needs remain unmet. Integrating psychological support into exercise rehabilitation or PA promotion programs may address this gap. Some patients explicitly reported that PA itself is a means of emotion regulation, which is consistent with previous findings (Bermudo-Gallaguet et al., 2025; Chen et al., 2026). Therefore, providing enjoyable activities that help patients focus on the positive experiences of PA may facilitate increased engagement.

Regarding the perception of the long-term benefits of PA, this study revealed a specific manifestation among young and middle-aged adults: some participants directly associated sustained PA with fulfilling family roles and resuming work responsibilities. This finding endows the “future health valuation” dimension of the intertemporal decision-making ability model with social role connotations, reflecting the influence of China’s collectivist culture, which may differ from that of individualistic cultural contexts. In Western individualistic societies, identity-driven motivations for PA participation may be more closely related to the restoration of autonomy and self-esteem (Andersen et al., 2026; Carless and Douglas, 2008). These findings suggest that incorporating identity-role-related value assessment items into future measurement tools or intervention protocols may improve intertemporal decision-making ability and PA adherence in young and middle-aged stroke patients. However, this hypothesis is proposed based on a collectivist cultural context and requires further validation across different cultural settings.

In terms of future-oriented thinking, participants with better intertemporal decision-making ability generated positive and specific future scenarios (e.g., becoming a fitness influencer, returning to marathon racing) and constructed causal chains linking “current actions to future outcomes” to facilitate PA engagement and adherence. This is consistent with previous findings that episodic future thinking (i.e., the human ability to mentally project oneself forward in time and pre-experience possible future events) positively influences PA (Atance and O’Neill, 2001; Giacobbi et al., 2018). Notably, some patients generated “warning imagery” (e.g., being bedridden in the future without anyone to care for them), which, although temporarily motivating, may impair long-term mental health (Wang and He, 2020). In future stroke health management, incorporating positive future-oriented thinking (e.g., generating goal possibilities, meaningful engagement, and episodic future thinking training) to enhance individuals’ access to positive expectations of future scenarios may help counteract negative emotions (Pillny et al., 2026). Additionally, some participants perceived the future as distant, which their age may explain. Previous studies (Bartels and Urminsky, 2011; Hershfield, 2011) have shown that younger individuals tend to have weaker future self-continuity, i.e., a weaker psychological connection between the present and future selves, which, in turn, leads them to perceive the future as more distant. Fortunately, participants adopted “small and proximal” goal setting and plan visualization to narrow the perceived distance to the future. Future empirical research could further explore the effect of goal-setting preferences on patients’ intertemporal decision-making ability.

### 4.1 Implications of the study for practice

(1) Development of assessment tools: Given the multidimensional nature of intertemporal decision-making ability in PA contexts among young and middle-aged stroke patients, future efforts could develop specific measurement tools to identify weaknesses in patients’ decision-making trade-offs regarding PA, thereby providing a basis for individualized interventions.
(2) Developing personalized intervention protocols: For participants with poor impulse control, set immediate rewards, lower the baseline level of activity, and mobilize positive experiences might help them overcome the impulse toward physical inactivity; for patients with difficulties in emotion regulation, healthcare providers could guide them to use attentional shifting, positive psychological cues, and focus on positive emotional experiences during PA. For participants who underestimate the future value of PA, healthcare providers could use motivational interviewing and risk communication to emphasize its future value. Furthermore, incorporating family narratives and expressions of role responsibility during the interview may be helpful. For patients with deficient future-oriented thinking, using episodic future imagination to guide patients toward positive imagery might reduce the psychological distance to the future. Importantly, these recommendations are derived from patient narratives in qualitative research and lack direct evidence; therefore, they require further validation in empirical studies with larger sample sizes.

### 4.2 Limitations

This study has several limitations. First, it is a single-center study with a sample limited to community-dwelling young and middle-aged stroke patients with mild disability. Consequently, the generalizability of our findings to patients with severe functional impairment is limited. They often face greater barriers to PA and may exhibit different intertemporal decision-making patterns. Future research should include patients with varying levels of functional impairment to explore the influence of functional status on intertemporal decision-making ability. Furthermore, the participants had relatively high educational levels, which limits the generalizability of the findings to patients with lower health literacy or those in rural areas. The classification of marital status was primarily married/unmarried, failing to capture differences in the impact of other statuses, such as divorce, on ability performance. Future sampling should adopt more detailed marital categories and perform subgroup comparisons. Second, information on participants’ lesion locations, neuropsychological status, and executive functions was not collected, which may potentially affect intertemporal decision-making, impulse control, and future-oriented thinking. Future research should integrate neuroimaging and neuropsychological assessments (e.g., executive function tests) to further explore the influence of neurological and cognitive functions on decision-making abilities in stroke patients. Third, this study was conducted within the Chinese cultural context, and the conceptual model may exhibit cultural specificity under collectivist norms, which limits its generalizability to other cultures. Future cross-cultural validation is therefore needed. Finally, the use of a predefined interview framework may have limited the emergence of new content, although this study employed open-ended questions and a combination of deductive and inductive approaches to minimize this bias. Additionally, recall bias may have influenced the interviews. Future research could conduct longitudinal interviews at different stages of the disease to capture a more realistic decision-making process.

## 5 Conclusion

This study employed a qualitative research approach to construct a conceptual model of intertemporal decision-making ability within PA among young and middle-aged stroke patients. The model reveals a multidimensional structure comprising impulse control, emotion self-regulation, future health valuation, and future-oriented thinking and planning. Moreover, the distinctive age-related identity enriches these dimensions with social role connotations. This conceptual model is expected to provide insights for the development of specific assessment tools and targeted intervention strategies.

## Data Availability

The datasets generated and/or analysed during the current study are not publicly available due to the confidentiality of participants, but are available from the corresponding authors upon reasonable request.
