# Supplementary material for: Development of a conceptual model of intertemporal decision-making ability for young and middle-aged stroke patients within physical activity: a qualitative study
Source: Front Psychol. 2026 Jul 20;17:1877769. doi: 10.3389/fpsyg.2026.1877769 (PMC13429451; doi:10.3389/fpsyg.2026.1877769)
Supplement: Supplementary file 1 [file Data_Sheet_1.pdf]

Supplementary file 1 Example of the coding process

Participants' quotation	Coding	Subtheme	Theme
"I can usually only persist for 20 minutes, and then I feel tired and cannot continue" (P1)	Fatigue triggers urge to stop	Impulse awareness	Impulse control
"I tell myself, if I complete 80 reps today, I'll give myself a reward, something I've wanted to eat but haven't..." (P12)	Self-reward to overcome inactivity	Inhibition of immediate impulses	Impulse control
"If I exercise for one or two weeks and see no results, I feel anxious and have thoughts of not wanting to exercise." (P5)	Positive- feedback- deficit anxiety and physical activity motivation loss	Emotional awareness	Emotional self-regulation
"I listen to music to distract myself. That is, I focus on the movements rather than on my emotions, and that makes me feel better" (P1)	Emotion regulation through attentional shift	Application of emotion regulation strategies	Emotional self-regulation
